# Size- and Time-Dependent Particle Removal Efficiency of Face Masks and Improvised Respiratory Protection Equipment Used during the COVID-19 Pandemic

**DOI:** 10.3390/s21051567

**Published:** 2021-02-24

**Authors:** Anja Pogačnik Krajnc, Luka Pirker, Urška Gradišar Centa, Anton Gradišek, Igor B. Mekjavic, Matej Godnič, Metod Čebašek, Tina Bregant, Maja Remškar

**Affiliations:** 1 Jožef Stefan Institute, Jamova Cesta 39, 1000 Ljubljana, Slovenia; anja.pogacnik@ijs.si (A.P.K.); urska.gradisar@ijs.si (U.G.C.); anton.gradisek@ijs.si (A.G.); igor.mekjavic@ijs.si (I.B.M.); maja.remskar@ijs.si (M.R.); 2 Novo Mesto General Hospital, Šmihelska Cesta 1, 8000 Novo Mesto, Slovenia; matej.godnic@sb-nm.si; 3 HYLA d.o.o., Brnčičeva Ulica 47, 1231 Ljubljana, Slovenia; metod.cebasek@hyla.si; 4 CIRIUS, Novi trg 43 a, 1241 Kamnik, Slovenia; tina.bregant@gov.si; 5 Faculty of Mathematics and Physics, University of Ljubljana, Jadranska Cesta 19, 1000 Ljubljana, Slovenia

**Keywords:** COVID-19, filtration efficiency, personal protection equipment, surgical mask, textile mask

## Abstract

Size- and time-dependent particle removal efficiency (PRE) of different protective respiratory masks were determined using a standard aerosol powder with the size of particles in the range of an uncoated SARS-CoV-2 virus and small respiratory droplets. Number concentration of particles was measured by a scanning mobility particle sizer. Respiratory protective half-masks, surgical masks, and cotton washable masks were tested. The results show high filtration efficiency of FFP2, FFP3, and certified surgical masks for all sizes of tested particles, while protection efficiency of washable masks depends on their constituent fabrics. Measurements showed decreasing PRE of all masks over time due to transmission of nanoparticles through the mask-face interface. On the other hand, the PRE of the fabric is governed by deposition of the aerosols, consequently increasing the PRE.

## 1. Introduction

The first line of defense for the widespread and rapid droplet and airborne transmission of the Sudden Acute Respiratory Syndrome Coronavirus 2 (SARS-CoV-2), which resulted in the Coronavirus disease 2019 (COVID-19) pandemic, is the protection of the airways using appropriate oronasal masks. The key characteristic of such face masks is their particle removal efficiency (PRE) from the inspired (protecting the wearer) and expired air (providing protection for those in close proximity of the user). In spring 2020, the factors limiting the production of masks were the short supply of nonwoven polypropylene fabric, a key component of the masks’ filter, as well as the disruption of international trade, with China being by far the largest producer of personal protective equipment [1]. The industry worldwide has since rebound; however, there is a tremendous variation in the quality and appropriateness of available products, primarily due to the lack of standardization for all types of protective masks [2] and an increased demand for testing at certification institutions, which were ill-prepared and not equipped to handle this increased demand. As the main mode of transmission between individuals is through respiratory droplets (aerosols) generated by breathing, sneezing, coughing, as well as speaking [3,4,5,6], face masks are considered one of the most efficient means of reducing transmission risk, not only for healthcare workers, but also for the general population [7].

Specific situations call for the use of specific types of face masks. Surgical masks are typically used by healthcare providers. These masks are mainly intended to catch the liquid droplets from the wearer’s mouth and nose with its inner absorbing layer, as well as to prevent the entry of droplets from outside. They do not fit tightly on the face, but nevertheless provide some degree of protection of the wearer from external aerosol particles. Filtering facepiece respirators (FFR), such as FFP2 and FFP3 (European designation), N95 (US), KN95 (China) or P2 (Australia and New Zealand) masks offer a higher degree of protection by filtering majority of the aerosol particles. They are designed to fit tightly on the face. These masks are used in higher-risk environments, especially in healthcare settings when highly contagious airborne viruses and bacteria could be encountered. Another type of mask used in high-risk environments are the elastomeric respirators that are also available in full-face designs. Surgical masks and FFRs are also available in combination with additional filters. The most commonly used filters contain activated carbon for filtration of toxic gases such as CO_x_, SO_x_, NO_x_, pollens, odors, and other pollutants [8]. All the above masks are classified as personal protective equipment (PPE) and must adhere to a specific set of standards. In Europe there are three standards that describe the protocols on how to test medical masks [9], filtering half masks [10], and particle filters [11]. The penetration of particles through a mask is measured according to European Standard EN 13274-7 [12]. Masks are evaluated for a range of characteristics; the most common being breathability (meaning that the wearer should be able to breathe comfortably while wearing the mask), particulate filtration, bacterial filtration, as well as fluid resistance and flame resistance [13]. The present study focusses on filtration efficiency. The PRE, also called the particulate filtration efficiency (PFE) is defined as the ratio of the particles detected upstream and downstream of the mask [13]. Such tests are performed using 0.1 µm polystyrene latex particles which have been suspended in water, and the aerosols were created by means of a particle generator. An alternative method uses NaCl aerosol where the particle diameters are between 10 nm and 10 µm (average diameter of 300 nm). For bacterial filtration, Staphylococcus aureus is typically used (size ranges from 3.0 ± 0.3 µm). For the evaluation of viral filtration performance, which is not a standard test protocol, different types of viruses are used [2,13].

As the COVID-19 pandemic caught the world unprepared, several issues arose a during the crisis, including the use of appropriate PPE. Knowledge regarding the transmission of the SARS-CoV-2 virus is accumulating every day and it calls for an agile and adapting (changed) response. During the first months of the pandemic, it was recommended that the use of facepiece respirators and surgical masks should be reserved for healthcare workers to prevent the shortages for this group of individuals, since they are at highest risk of exposure [14]. The recommendation for the general public was typically to wear cloth masks [2,15]. Later, with the increased production, surgical masks became available for the general public, as well. Cloth masks are typically made out of two or more layers of cotton fabric or of other material [16,17]. As opposed to personal PPE, there are no specific standards regarding cloth masks. Nevertheless, apart from allowing the wearer to breathe comfortably (breathability) and being made from non-toxic materials, it is expected that these masks have an appropriate particle filtration efficiency.

Using a scanning mobility particle sizer, we performed particle removal efficiency measurements of professional, home-made, and improvised PPE, focusing on the size- and time-dependent filtration efficiency of aerosol particles ranging from 12.2 to 572.5 nm. The diameter of uncoated SARS-CoV-2 viruses ranges from 50 to 200 nm [18] which makes the evaluation of PRE in the appropriate range of particle diameter an important factor.

During the first months of the COVID-19 pandemic, the lack of PPE provided a fierce impetus for research into the filtration efficiency of different cloth masks. Lustig et al. [16] compared the filtration efficiency of over 70 different fabric combinations by tracking the transmission of virus-like nanoparticles through the materials. Outward aerosol particle emission from different home-made masks was also studied [15]. Quantitative methods for comparative assessment of particle removal efficiency of fabric masks were developed and the particle removal efficiencies were between 28% and 91% [17]. The filtration efficiency of household materials could be enhanced by triboelectric charging [19]. The filtration process depends not only on the type of fabric used in the mask, but also on the number of fabric layers and the structure of the fabric threads [2,15]. For the purpose of testing the filtration efficiency of masks, a variety of experimental procedures were proposed. Aerosols typically used were NaCl [19] and KCl [20] particles, aerosol particles produced by live subjects [15], composite nanoparticles [16], and latex spheres [21].

In the present study we investigated the particle removal efficiency of masks using an approach described previously [22]. Size- and time-dependent PRE of professional masks of types FFP2 and FFP3, different types of surgical masks, a series of cotton fabrics used for home-made masks, and some improvised personal protection equipment will be presented. Although PRE of certified masks is usually known and recommended wearing times are declared, size and time dependencies of the particle transmission are not known.

## 2. Materials and Methods

### 2.1. Particle Removal Efficiency Testing

**Figure 1:**
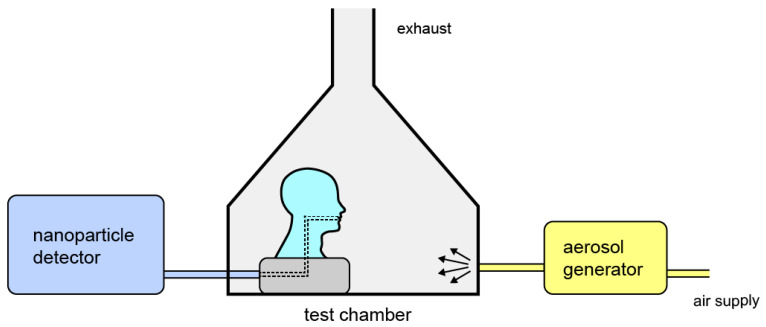
A schematic representation of the experimental setup. The arrows indicate the direction of the aerosol. A vacuum pump provided an inspiratory flow of 4 L.min^−1^ at the head manikin mouth.

The experimental arrangement for evaluating the PRE of masks is shown schematically in Figure 1. It consists of an aerosol generator (Topas SAG 410, Topas GmbH, Dresden, Germany), which disperses the aerosol standard powder [23] into the test chamber [24,25]. The test mask is donned on a standard head manikin [26] positioned within the test chamber. These manikins are used to evaluate the work of breathing of respirators by connecting the mouth of the manikin to a breathing simulator, and measuring the inspiratory and expiratory pressure during different levels of minute ventilation.

The mouth of the head manikin was connected to the nanoparticle detector providing a constant inspiratory flow of 4 L·min^−1^. The inspired air was sampled with a nanoparticle detector (Scanning Mobility Particle Sizer (SMPS), model 3080 L85; TSI Incorporated, Saint Paul, MN, USA) equipped with a desiccator, soft X-ray neutralizer, long differential mobility analyzer, and a water condensation particle counter (WCPC; model 3785; TSI Incorporated, Saint Paul, MN, USA). In this manner, we measured the nanoparticle (NP) total concentration (TC) and time-dependent size distribution, with 3-min time resolution. The sheath flow rate through the DMA was 4 L·min^−1^, while the aerosol flow rate through the WCPC was 1 L·min^−1^. No impactor was used upstream the neutralizer as it had no impact on the particle distribution, Figure S1. The SMPS was operated with the Aerosol Instrument Manager version 9.0.0.0, where the multiple charge correction and the diffusion correction were enabled. The inverted SMPS measurements were further analysed using our own software written in Mathematica.

The experimental procedure used in this paper is not fully in accordance with any filtration/mask testing national/regional/international protocol as it was developed as an urgent reaction to the demand for a practical mask assessment (using the equipment readily available) and not for actual accreditation purpose. According to the EU standards [9,10,11] and literature [13] the particulate filtration efficiency can be measured with a wide variety of particles such as 0.1-µm polystyrene latex particles or sodium chloride (NaCl) aerosols, where the PFE is determined either by counting the number of particles with an optical counter or measuring the concentration of the material with a flame photometer, upstream and downstream of the mask. During the tests various conditions and requirements need to be fulfilled such as the concentration of the particles, the size distribution of the particles, humidity and the airflow rate through the mask.

**Figure 2:**
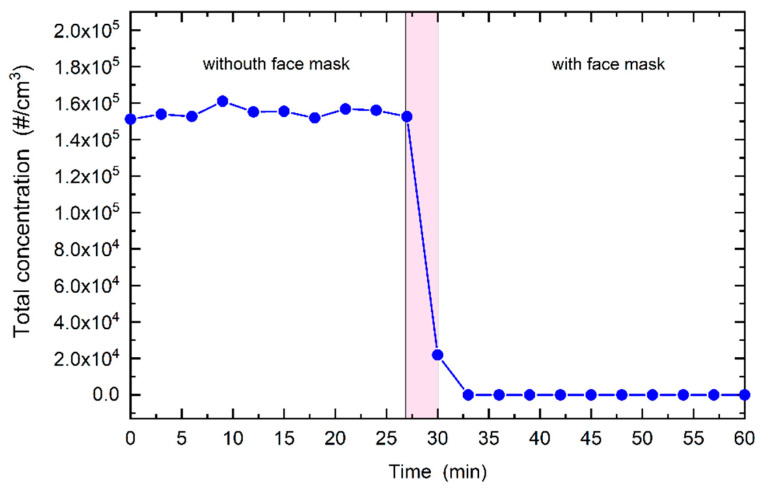
Total particle concentration as a function of time. Left side: Manikin head without the face mask; coloured area: The test chamber briefly opened and the face mask is mounted on the head manikin; right side: Manikin head with the face mask.

The electrical mobility diameter of counted nanoparticles was from 12.2 to 572.5 nm, which covers the size range of the SARS-CoV-2 virus, with size of around 50–200 nm in diameter in an uncovered state [18], and small respiratory droplets. Before each measurement, the test chamber was injected with the standard powder for 30 min, so that steady state conditions were obtained, as shown in Figure S2. The peak of the size distribution of aerosol particles was between 40 nm and 50 nm, Figure S3. To perform a measurement of PRE, the test chamber was briefly opened and the test mask was donned on the head manikin (the process took about 30 s and caused a minimal change in the particle concentration in the chamber as seen in Figure S2). An example of the temporal response of the normalized number concentration (d*N*/dlog*D*_p_ [#/cm^3^]) of NPs given as diameter in log scale vs. time during a typical measurement is shown in Figure S4. The normalized concentration, d*N*/dlog*D*_p_, measured with the SMPS, is the total concentration (d*N*) in within the measured range divided by the difference between the logarithms (dlog*D*_p_) of the lower and upper diameter of the counted particles. This makes the normalized concentration independent of the channel width of the instrument [27]. The normalized concentration in this form is used so that results from instruments with different channel widths can be compared. Each measurement lasted 30 min. The time evolution of the total concentration during a measurement is shown in Figure 2. Before the test chamber is opened, the TC is almost constant, indicating a high degree of stability of the system. Based on the definition of the PRE as the ratio of the concentration of particles that are detected when a mask is donned on the manikin [13] relative to the concentration of particles detected when no mask covers the head manikin’s mouth, the overall PRE is determined as:

(1) $$PRE=1-\frac{TC_{i,\mathrm{ON}}}{{\bar{TC}}_{\mathrm{OFF}}}$$

where $TC_{i,\mathrm{ON}}$ is the total concentration, when the mask is on the head manikin, and ${\bar{TC}}_{\mathrm{OFF}}$ is the average total concentration without the mask. $TC_{i,\mathrm{ON}}$ was determined from the third measurement as it took approximately 6 min for the particle concentration to stabilize inside the chamber.

The size-dependent PRE (SPRE) is calculated according to:

(2) $$SPRE=1-\frac{{\left[\frac{dN}{d\log D_{p}}\right]}_{i,\mathrm{ON}}}{{\bar{\left[\frac{dN}{d\log D_{p}}\right]}}_{\mathrm{OFF}}}$$

where ${\left[\frac{dN}{d\log D_{p}}\right]}_{i,\mathrm{ON}}$ is the normalized concentration, d*N*/dlog*D*_p_, of the third measurement with the mask on the head manikin with particle diameter *D*_p_. The ${\bar{\left[\frac{dN}{d\log D_{p}}\right]}}_{\mathrm{OFF}}$ is the average value of the normalized concentration, d*N*/dlog*D*_p_, when there is no mask on the artificial head at the particle diameter *D*_p_.

The time-dependent PRE was calculated using Equation (1), where $TC_{i,\mathrm{ON}}$ is the total concentration at each time point. PRE given as a particle diameter in log scale vs. time of measurement plots were calculated with Equation (2) where the PRE was calculated in each time point for each particle diameter.

Particle removal efficiency was determined separately for the masks, and for the material from which the masks were made. The mask’s PRE is affected by the filtration efficiency of the material and the design of the mask [28]. Namely, a mask that does not fit properly on the face will allow particles to pass through the mask, either through the material or by way of the gaps between the mask-face interface, respectively [29,30]. For a well-designed mask, the mask PRE will almost equal the material PRE. The material PRE was determined using a special holder carrying 4 cm × 4 cm large piece of material at wider part and connected with a tube to the mouth of the head manikin. This design prevented any leakages and ensured that the surface exposed to the particulate matter was always the same.

### 2.2. Selection of Masks

The CE labelled respiratory face masks were manufactured according to the European EN 149:2001 + A1:2009 standard. The FFP3 masks require at least 99% filtration efficiency of aerosol particles, while the FFP2 masks require at least 94% filtration efficiency. For FFP3 mask, the inward leakage should be less than 2% and for FFP2 masks less than 8%. Surgical masks used in the experiment were manufactured according to the European EN 14683:2019 standard. The cotton fabrics for making washable masks were tested in two layers. An off-the-shelf full-face snorkeling mask and a modified professional gas mask were tested. The reproducibility of the method was performed on the FFP2 and FFP3 masks, with measurements of three different sections of the material as published in our previous work [22]. Due to negligible variations of filtration performance among identical masks, the rest of the measurements were carried out in a single repetition.

### 2.3. Scanning Electron Microscopy

Scanning electron microscopy (SEM) images were made with a Helios NanoLab 650 Focused Ion Beam-scanning electron microscope. Each sample was coated with approximately 10 nm of carbon to prevent charging effects during electron irradiation. The SEM was used to analyze the size and structure of the fibers of cotton masks.

## 3. Results and Discussion

### 3.1. Size-Dependent PRE Measurements

#### 3.1.1. FFP2 and FFP3 Masks

**Figure 3:**
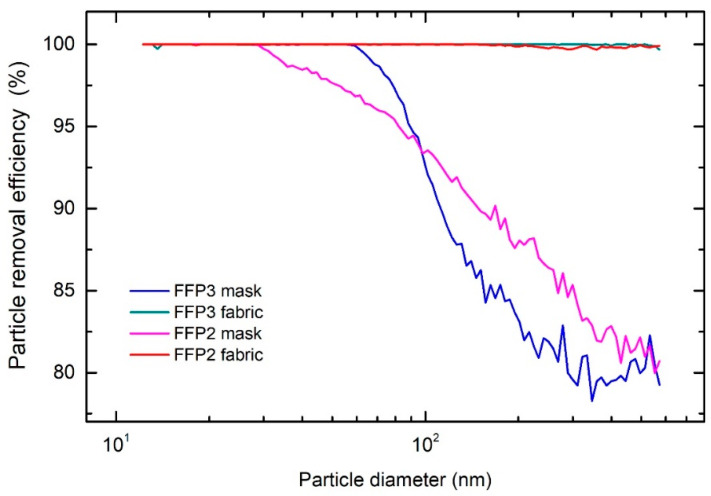
Particle removal efficiency as a function of particle diameter for the FFP2 and FFP3 respiratory masks and their fabrics.

**Figure 4:**
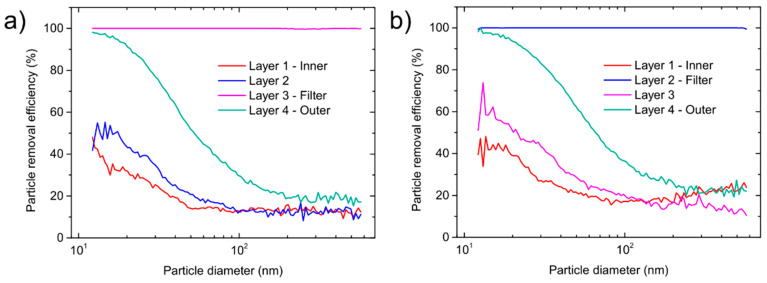
Particle removal efficiency as a function of particle diameter for single layers: (**a**) FFP2 mask; (**b**) and FFP3 mask.

The overall PRE for the FFP2 mask was 96% and 97% for the FFP3 mask. The size-dependent PRE for both type of masks is shown in Figure 3. For the FFP2 mask, the PRE starts to decrease at sizes above approx. 40 nm, while for FFP3 a reduction occurs for particles above 60 nm. The decrease in PRE is attributed to the air bypassing the face mask filtering material due to a less-than-perfect fit to the head manikin, which is a common problem in FFR testing [17,28]. As expected, the overall values of PRE of the fabrics were higher (Figure 3). The PRE for the FFP2 fabric was above 98.6%, while the PRE of FFP3 fabric was above 99.9%. Both materials filter out most of the particles throughout the whole measuring range (12.2 to 572.5 nm). As previously shown [22], only one layer inside such type of masks acts as a filter and contributes to the high PRE, while other layers have other functions such as structural support [13]. The filtering layer is composed of various fibers with diameters ranging from 500 nm up to 10 µm, while supporting layers typically have fibers with diameters between 10 µm up to 100 µm [22]. To elucidate contributions of different layers, size-dependent measurements were performed on individual layers composing the FFP2 and FFP3 masks. The results shown in Figure 4 reveal that the layer 3 in the FFP2 mask and the layer 2 in the FFP3 mask contributed the most to the high PRE. All other layers have substantially lower PRE values (presented in Table 1) and reflect their non-filtering role in the mask composition.

#### 3.1.2. Surgical Masks

At the onset of the COVID-19 pandemic, manufacturers and suppliers could not immediately meet the worldwide demands for certified masks. Consequently, the market was flooded with different types of masks labelled as surgical, but of questionable quality, and lacking any certification. To demonstrate such assessment, we present the results of four different surgical masks, manufactured according to the following certificates: Mask A—EN 14683, type II R; mask B—EN 14683, type II; mask C—EN 14683:2019; mask D—no certificate was provided.

**Figure 5:**
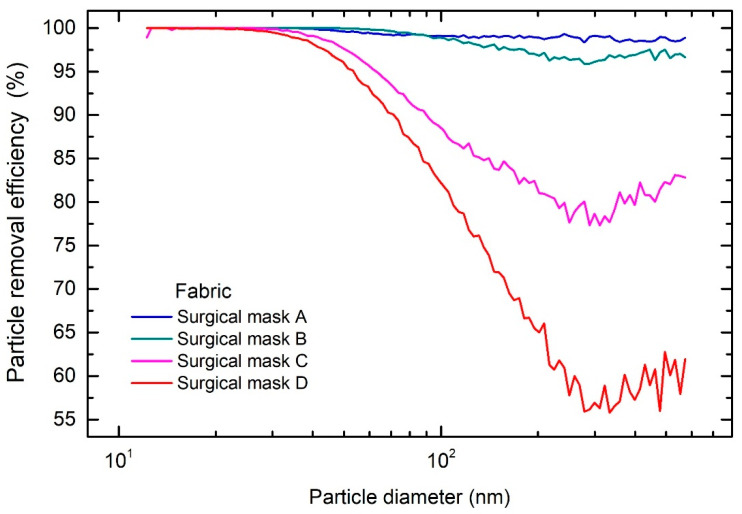
Particle removal efficiency as a function of particle diameter for the fabrics of different types of surgical masks: A-EN 14683, type II R; B-EN 14683, type II; C-EN 14683:2019; D—without certificate.

The measured overall PRE of the masks shows similar size-dependent particle removal efficiencies, as shown in Figure S5. The overall PRE for the surgical masks donned on the head manikin was between 65% and 80%. Surgical masks do not provide a good fit on the face [28], unlike FFP2 and FFP3 masks. Therefore, only the PRE of their fabrics was investigated, cf. Figure 5 for details. Fabric of mask A, which was composed of four layers, had the highest overall PRE of 99.5%. As seen from Figure 5, the fabric filters out most of the particles in the measured range, and has a comparable performance to the fabric of the FFP2 and FFP3 masks. Similarly, the fabric of mask B had the overall PRE of 99.4%. For the fabric of mask C, the overall PRE was 94.6%, which is in compliance with the lower standard class of the surgical mask [13]. The fabric of mask D had the lowest overall PRE of 91.5% and therefore should be used with caution, especially in high-risk environments.

The majority of the penetrated particles were in the range between 200 nm and 500 nm in diameter, which are too small for direct impaction, interception or settling and too big for diffusion and electrostatic attraction to be an efficient removal mechanism [27]. The static charge present of the fibers creates a non-uniform electrical field that imposes a force on the particles which have a net charge or an induced dipole. These forces then retard the progress of the particles, increasing the probability that they impact a fiber due to diffusion and/or directly attract them to the surface of the fiber. Such fibers, called electrets, are commonly used in modern FFRs. In the case of the SARS-CoV-2 virus a theoretical model predicts that electret fibers can successfully capture different viruses across a broad range of species and environments [31].

The different PRE of masks and their fabrics demonstrates the need to evaluate masks also from an ergonomics perspective. Namely, an optimal mask design is essential to ensure that the PRE characteristics of the mask approach that of the fabric. Health workers wear a mask for the duration of a work shift, and during this time, the layers of the mask will absorb the expired water vapor. The manner in which the added moisture in the particular layers affect the PRE is unknown, but it would probably lower the overall performance due to loss of charge [22]. The current tests were conducted at a constant inspiratory flow rate of 4 L·min^−1^ at which the SMPS was calibrated. Resting minute ventilation is more than double, and furthermore comprises an inspiratory and expiratory phase. Some recent studies already evaluated the effect of light to moderate work with different levels of minute ventilation [32,33]. Simulating the inspiratory and expiratory phases of respiration may also affect the mechanical structure of the mask layers. After several hours of use, the layers within the mask become physically more separated, which might affect the PRE of such masks.

Despite the observed differences in PRE of different surgical masks, their use by the general public as hygienic masks mitigating the spread of SARS-CoV-2 is advisable, because of their relatively high fabric PRE in the entire size range of the tested particles.

#### 3.1.3. Cloth Masks and Fabrics

Due to the inability of the manufacturers to meet the global demand for masks, a variety of uncertified cloth face masks appeared on the market. The so-called hygienic masks are made of different textiles with no standards regulating their performance. The efficiency of these masks as a means of protecting the user from viruses is not known. Cloth masks come in a wide variety of designs and compositions. It has been recommended [14] that a home-made cloth mask should comprise at least two layers of fabric. As the design and fit of the mask to the face can substantially influence the PRE performance (as demonstrated above for the surgical masks), the testing was performed only on the most common double-layer fabrics that used to make cloth masks due to their ability of washing and comfortable wearing.

**Figure 6:**
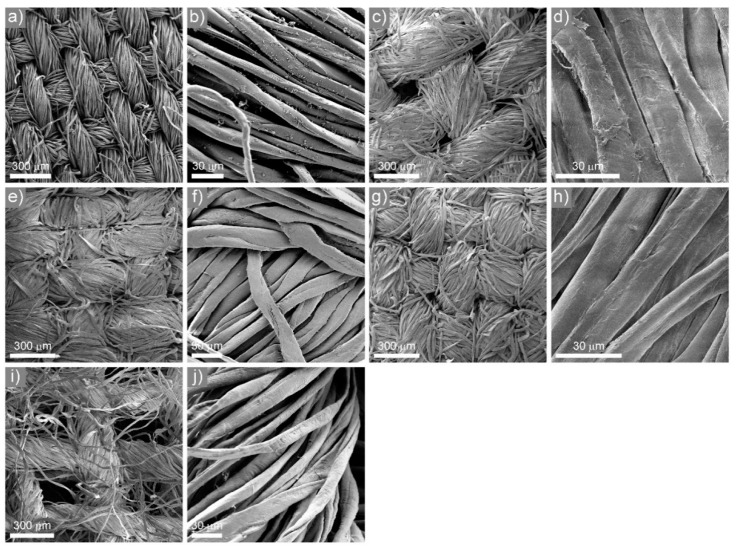
SEM images of: (**a**,**b**) Poplin A; (**c**,**d**) Poplin B; (**e**,**f**) Poplin C; (**g**,**h**) Poplin D; (**i**,**j**) Tetra.

SEM images of the five fabrics tested in this study are shown in Figure 6 at two magnifications. The fabrics differ in the shape and diameter of the threads as well as in the density of their weaving. B, C, and D poplin fabrics have threads with similar diameter and flattened shape, while threads in poplin A and tetra are narrower and of rounded cross-section. The density of waving is highest in A, C, and D poplin fabrics, slightly smaller in poplin B, and the lowest in tetra.

**Figure 7:**
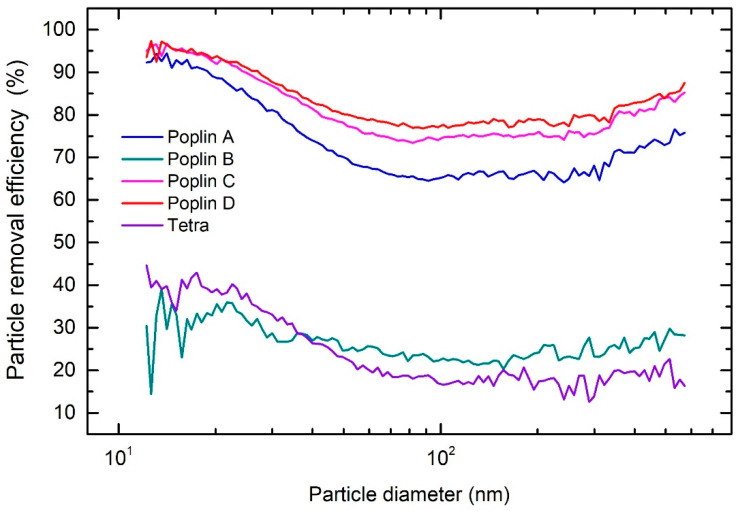
Particle removal efficiency as a function of particle diameter for tested fabrics.

The overall PREs of five cotton fabrics in comparison with FFP2, FFP3 and surgical masks are shown in Table 2. The overall PRE of A, C, and D poplin fabrics was over 70%, while for poplin B and tetra the PRE was below 30%. As can be seen from Table 2, one cannot infer the PRE from the fabric weight alone—the structure of the fabric and the size of the fibers are more important than the weight. The A, C, and D poplin fabrics have a similar size-dependent PRE, as shown in Figure 7. The PRE starts to decrease for particles larger than 20 nm, and drops down to 65% for fabric A. PRE is the lowest for particles between 100 nm and 300 nm, which is expected, as these particles are more difficult to filter from the air due to the large diameter of the fibers and lack of any static charge on the fibers. For poplin B and the tetra fabric, the overall PRE is considerably lower due to large pore sizes, as seen in Figure 6c,i), enabling aerosol particles to pass unhindered through the material. In contrary, the threads form a tight mesh in the A, C, and D poplin fabrics forcing the aerosol particles to pass between the fibers, increasing the probability of their deposition onto the surface of fibers or getting stuck between them. Although the overall PRE for the presented fabrics is lower than that of FFP and surgical masks, some textiles appear to provide a suitable level of protection for the general public.

#### 3.1.4. Improvised Respiratory Masks

The shortage of PPE at the onset of the pandemic resulted in an initiative to modify full-face snorkeling masks as respiratory protection for health workers. Modifications of these masks allowed the attachment of high efficiency medical breathing filters (Filta-GuardTM filter, PRE > 99.9%, which were readily available) on the inspiratory and expiratory outlets of the mask. Some health workers also started using gas masks (Scott M95), replacing the filters with medical-grade filters. These gas masks are normally used by firefighters and military personnel. The improvised PPEs (snorkeling and gas masks) were made using available products that are not originally intended to be used as medical PPE. As both masks cover the entire face, they were deemed a possible substitute for medical PPE. In both of these masks, the filter is disposable while the rest of the mask can be disinfected and thus reused. Both masks were modified with 3D printed adapters so that the inspired air passes through the breathing filter and the exhaled air through a one-way valve, as shown in Figure S6. As the adapter can be easily manufactured and the breathing filters are in ample supply, such masks were viewed as an attractive alternative and were even widely promoted on social and other media.

**Figure 8:**
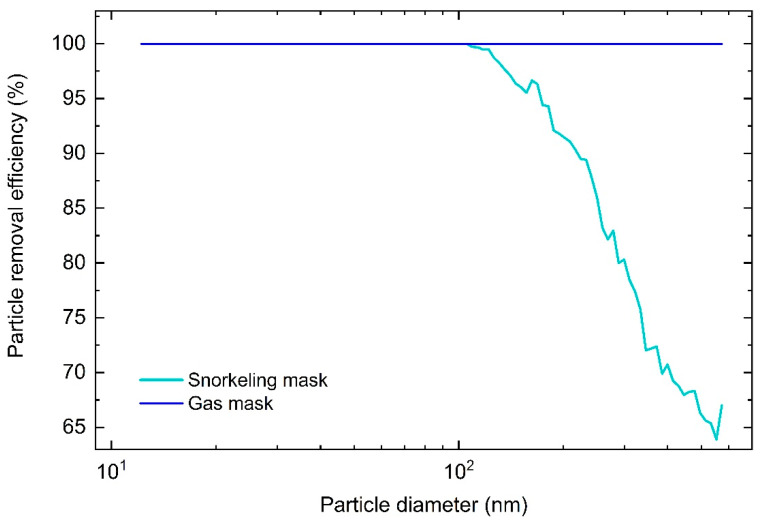
PRE as a function of particle diameter for the gas and snorkeling mask.

The overall PRE of the gas mask was the same as that of the breathing filter, indicating that there was no leakage. The snorkeling mask equipped with the breathing filter had an overall PRE of 96.8% which indicates some air leakage. This is evident from the size-dependent PRE graph, shown in Figure 8. The PRE for the modified gas mask is essentially 100% for all particle sizes, while for the modified snorkeling mask the PRE starts to drop at 100 nm and drops down to 65% at larger particle diameters, Figure 8. Although the overall PRE for the modified snorkeling mask is similar to a FFP2 mask, it should not be used in high-risk environments, as it does not provide suitable protection.

As both gas and snorkeling mask cover the entire face, the accumulation of CO_2_ and depletion of O_2_ within the masks has to be evaluated, especially in modified snorkeling masks where the dead-space volume could pose a risk due to a poor fit of the oronasal mask.

### 3.2. Time-Dependent Measurements

**Figure 9:**
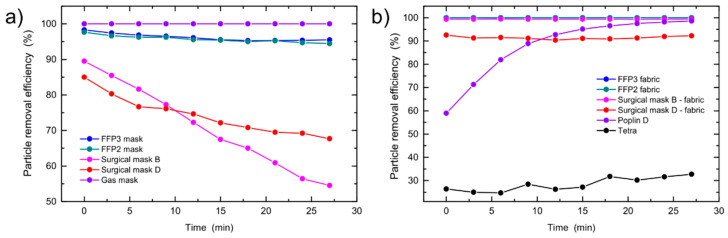
Particle removal efficiency (PRE) as a function of time for: (**a**) Face masks and (**b**) fabrics.

**Figure 10:**
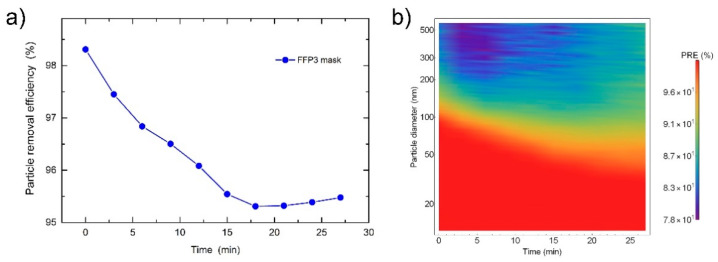
(**a**) Particle removal efficiency (PRE) as a function of time and (**b**) PRE given as a particle diameter in log scale vs. time of measurement plots for the FFP3 mask.

Although the filtration efficiency of the mask is usually given as a fixed number, it should be kept in mind that it changes with time due to various reasons. The time-dependent PRE measurements for different masks and fabrics are shown in Figure 9. PRE decreases with time for all masks, Figure 9a and Figure S7. From the PRE, given as a particle diameter vs. time in Figure S7 and Figure 10b), it is evident that the overall PRE decreases with time due to a decreased PRE for particles with smaller diameters. Although some studies [28] have shown that the size distribution of the penetrated particles through small leaks probes in FFRs does not change and is governed by the filtering material, at larger leak probes all particle sizes are equally represented. It has also been shown, that masks with higher filtration efficiency (such as FFRs) have a lower total inward leakage than masks with lower filtration efficiency (such as surgical masks) [28]. The particles penetrate mostly through the facepiece seal [33]. In FFRs, the face seal leakage-to-filter ratio is around 10 while for surgical masks is around 5 [33]. The decrease in the PRE for particles with smaller diameters most probably originates due to the particles that bypass the filtration material. When the mask was removed from the head manikin, trails of particles were observed on the head surface where they bypassed the mask. This could indicate that the thin layer of particles formed on the surface of the head manikin, screening the surface charge of the head manikin, allowing for more particles to enter the inlet leading to the nanoparticle detector. As the electrostatic charge affects mostly smaller particles [22,27], only the PRE for particles between 50 and 300 nm drops considerably over time, as it can be seen from Figure S7 and Figure 10.

**Figure 11:**
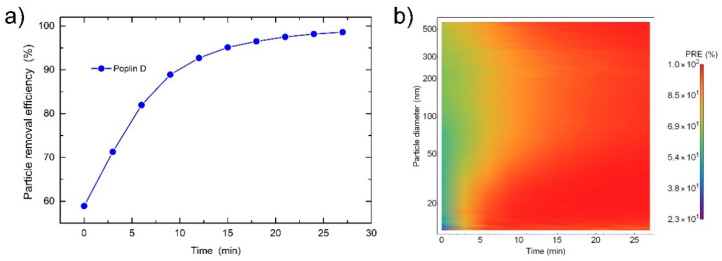
(**a**) Particle removal efficiency (PRE) as a function of time and (**b**) PRE given as a particle diameter in log scale vs. time of measurement plots for the Poplin D fabric.

On the other hand, the PRE of the filtration material usually increases with time, Figure 9b and Figure S8. This is attributed to the deposited aerosol on the surface of the mask, which acts as an additional barrier for other particles. An increase in PRE for almost 40% was observed on the Poplin D fabric, Figure 11. Although this means that a dustier mask filters particles better than a new one, the barrier also lowers the air permeability [34].

**Figure 12:**
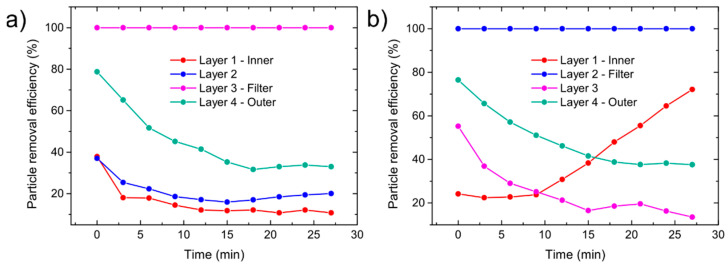
Particle removal efficiency (PRE) as a function of time: (**a**) FFP2 filtering material and (**b**) FFP3 filtering material.

Time-dependent measurements were also performed on individual layers of FFP2 and FFP3 masks. For both filtration layers, layer 2 for FFP3 and layer 3 for FFP2, the PRE was almost constant during the measurements and did not fall under 99.9%, Figure 12. As for the other layers, the PRE drops with time, probably due to charge screening. The polypropylene fibers used in masks usually have a surface charge. Such fibers are called electrets and the surface charge arises due to the trapping of charge carriers at defects during the manufacturing process or it is applied on finished respirators, commonly with the corona-discharge method [35,36,37]. With the accumulation of particles on the surface of the thicker fibers the charge is screened and the PRE drops, especially for particles with smaller diameter, which can be seen on the PRE given as a particle diameter vs. time in Figures S9 and S10. A noticeable increase in PRE occurred only in the inner layer of the FFP3 mask. This layer is composed of smaller fibers and a tighter mesh as other supporting layers, shown previously [22]. The PRE starts to increase due to the accumulation of dust particles, clogging the air pathways and artificially increasing the PRE.

It should be stressed that the fit factor of a mask is of great importance, as there is a strong association between fit factors and protection factors [38]. Although FFRs have an overall higher PRE than surgical or cloth masks, they can induce discomfort and lower wearing adherence due to an increased facial skin temperature, when compared to the medical surgical masks [39]. Although the overall PREs for fabrics and materials found in surgical masks were above 90%, the inward aerosol leakage around the edges of surgical face mask lowers the overall PRE for more than 10%. Our research coincides with resent CDC recommendations that suggests wearing a fabric face mask over a disposable surgical mask in order to ensure a better fit and therefore a better protection against COVID-19 [40].

## 4. Conclusions

Wearing respiratory masks is considered one of the most effective preventive measures against the spread of the SARS-CoV-2 virus. A wide variety of materials used in masks and different mask models revealed many knowledge gaps related to the mechanisms of aerosol filtration. The overall efficiency of different face masks and their fabrics in the size range of an uncoated SARS-CoV-2 virus and small respiratory droplets was tested by a scanning mobility particle sizer and their size- and time-dependent PRE was calculated. The experimental procedure was developed as an urgent reaction to the demand for a practical mask assessment using readily available equipment. The PRE for the FFP2 fabric was above 98.6%, while the PRE of FFP3 fabric was above 99.9% as both materials filter out most of the particles throughout the whole measuring range. Similar or slightly lower PRE values were observed for tested materials obtained from different surgical masks. Although the PRE of the material is similar in FFRs and surgical masks, the overall PRE of the mask itself differs from 15% to 31%. The lower PRE of the surgical masks is mainly due to the face–mask interface leakage as surgical masks are not intended to have a tight fit when worn. Different cotton materials that are usually used in cotton masks had a PRE between 26% and 82%. As expected, the PRE of cotton fabrics were lower than the PRE of materials that compose FFRs and surgical masks. This is mainly due to large diameter of the cotton fibers which also lack of any static charge. The large differences between different cotton materials come from the type of mesh, used to thread the fibers. Cotton materials with a tight mesh have a higher PRE than those with large pore sizes. Two different improvised respiratory masks were also tested where the PRE of the gas mask was the same as that of the breathing filter, indicating that there was no leakage while the snorkeling mask equipped with the breathing filter had an overall PRE of 96.8% which indicated some air leakage. Time-dependent PRE measurements were performed on different masks and materials. The PRE of the filtration material usually increases with time and is attributed to the deposited aerosol on the surface of the material, which acts as an additional barrier for other particles. Although this means that a dustier mask filters particles better than a new one, the barrier also lowers the air permeability, which may have a negative effect on the mask usability. On the other hand, the PRE decreases with time for all masks. In our experimental setup, a thin layer of dust formed on the head manikin, screening the surface charge and thus decreasing the PRE. In a real-life situation, the result could be different as the artificial head manikin is replaced with the wearers head and parameters such as humidity and airflow through the mask likely play an important role.

## Figures and Tables

**Table 1:** Particle removal efficiency (PRE) of different layers in the FFP2 and FFP3 filtering facepiece respirators (FFR)**.**

	Particle Removal Efficiency (%)				
	Layer 1 (Inner layer)	Layer 2	Layer 3	Layer 4 (Outer layer)	All Layers
FFP2	17.9	22.3	100.0	51.7	100.0
FFP3	22.8	100.0	29.0	57.1	100.0

**Table 2:** Overall particle removal efficiencies of different face masks and filters.

		PRE	
Type	Mask/Fabric	Mask	Fabric
FFP	FFP2	96.0%	98.6%
	FFP3	97.0%	99.9%
Surgical mask	A	79.9%	99.5%
	B	81.6%	99.4%
	C	65.8%	94.6%
	D	76.7%	91.5%
Cotton mask	Poplin A, 120 gsm	/	72.5%
	Poplin B, 110 gsm	/	26.0%
	Poplin C, 130 gsm	/	79.7%
	Poplin D, 130 gsm	/	82.0%
	Tetra, 185 gsm	/	24.7%
Gas mask		99.9%	/
Snorkeling mask		96.8%	/

## Data Availability

The data presented in this study are openly available on FigShare at https://doi.org/10.6084/m9.figshare.14079743.v1, accessed on 12 February 2021.

## Supplementary Materials

The following are available online at https://www.mdpi.com/1424-8220/21/5/1567/s1.
