# Supplementary material for: Size- and Time-Dependent Particle Removal Efficiency of Face Masks and Improvised Respiratory Protection Equipment Used during the COVID-19 Pandemic
Source: Sensors (Basel). 2021 Feb 24;21(5):1567. doi: 10.3390/s21051567 (PMC7956512; doi:10.3390/s21051567)
Supplement: Supplementary file 1 [file sensors-21-01567-s001.pdf]

# Size- and time-dependent particle removal efficiency of face masks and improvised respiratory protection equipment used during the COVID-19 pandemic

**Anja Pogačnik Krajnc[1,+], Luka Pirker[1,+,*], Urška Gradišar Centa[1], Anton Gradišek[1], Igor B. Mekjavić[1], Matej Godnič[2], Metod Čebašek[3], Tina Bregant[4], Maja Remškar[1,5]**

[1] Jožef Stefan Institute, Jamova Cesta 39, 1000 Ljubljana, Slovenia
[2] Novo Mesto General Hospital, Šmihelska Cesta 1, 8000 Novo Mesto, Slovenia
[3] HYLA d.o.o., Brnčičeva ulica 47, 1231 Ljubljana, Slovenia
[4] CIRIUS, Novi trg 43 a, 1241 Kamnik, Slovenia
[5] Faculty of Mathematics and Physics, University of Ljubljana, Jadranska cesta 19, 1000 Ljubljana, Slovenia
[+] These authors contributed equally
[*] Correspondence: luka.pirker@ijs.si

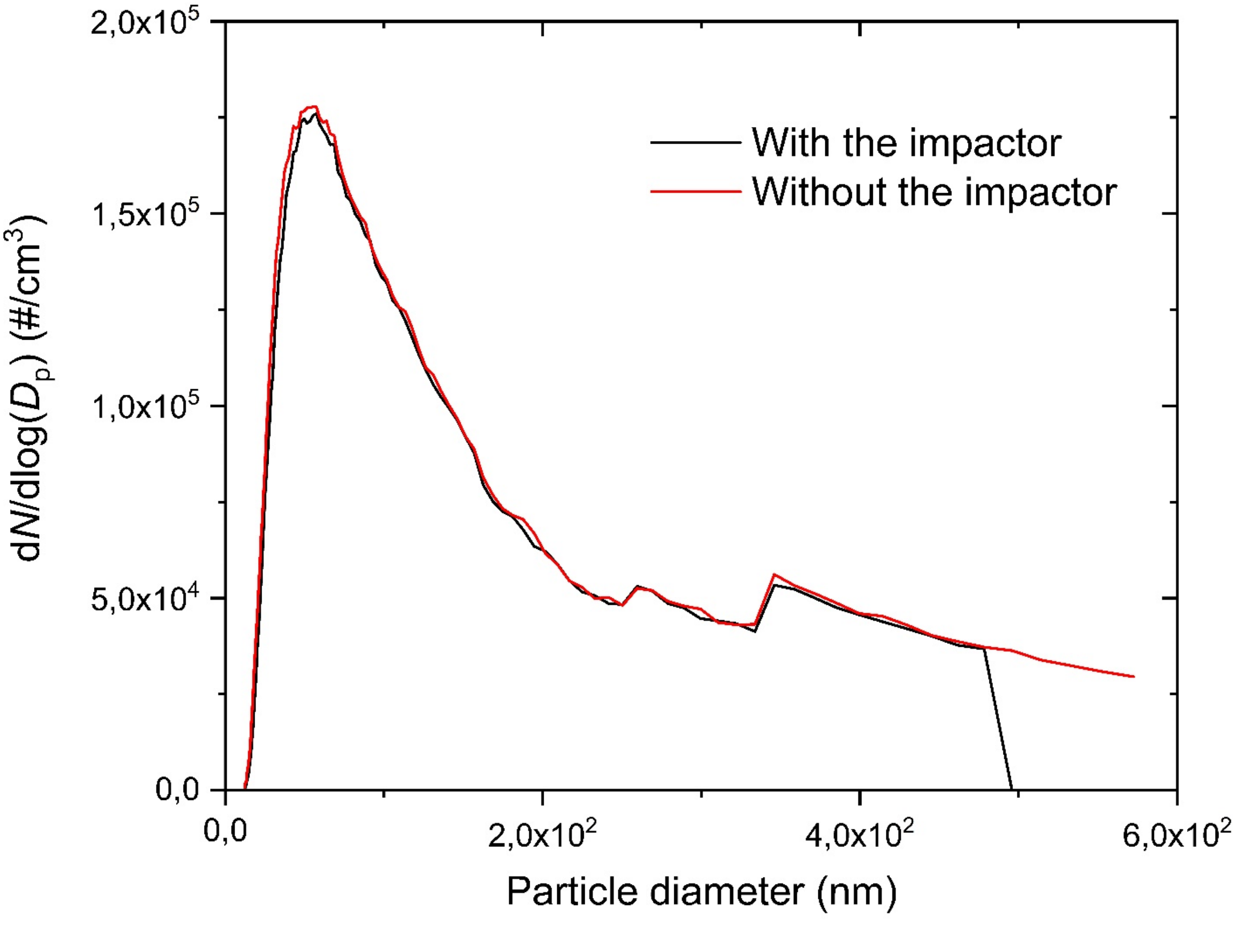


Figure 1S: Particle distribution ($dN/d\log D_P$) as a function of particle diameter $D_P$ with and without an impactor upstream the neutralizer.

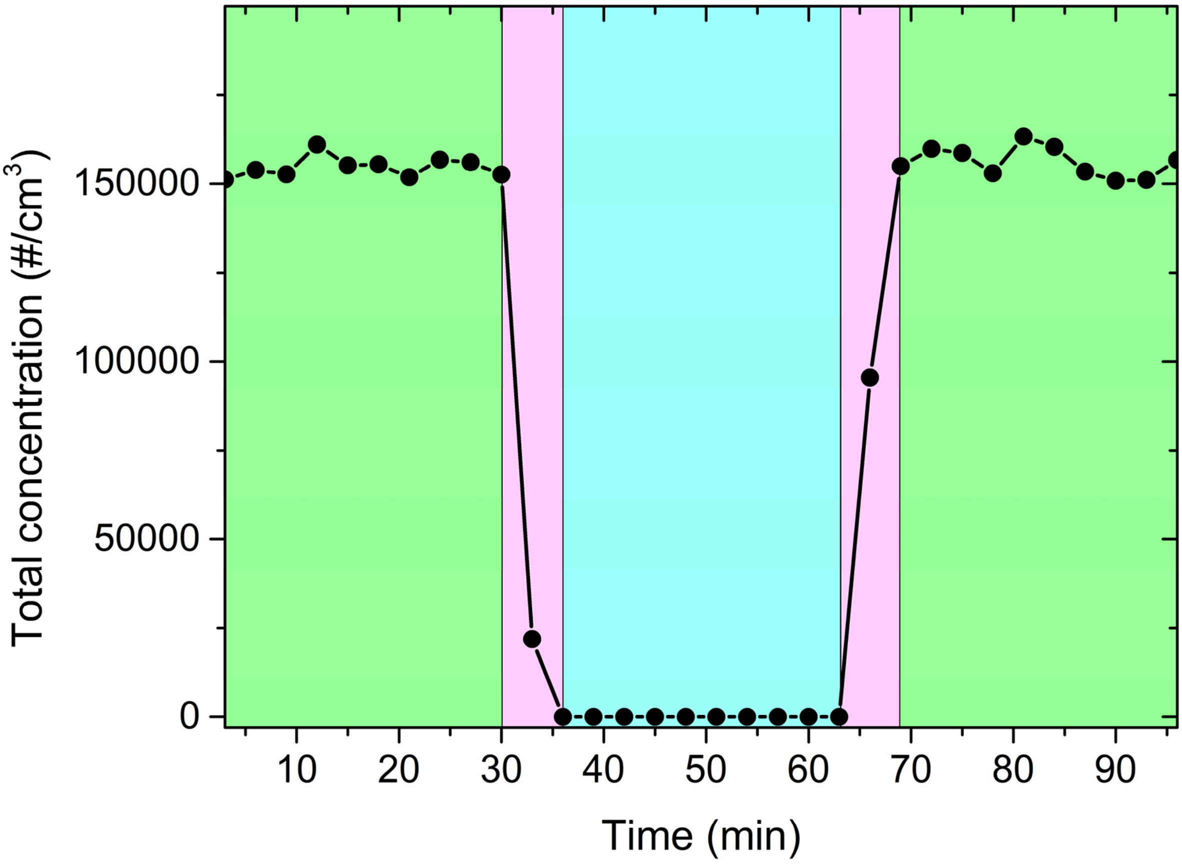


Figure 2S: Total concentration as a function of time during a measurement. The green area represents measurements without the mask/fabric, the purple area represents the measurement, when the mask/fabric is mounted on the artificial head, the blue area represents the measurement with the mask/fabric.

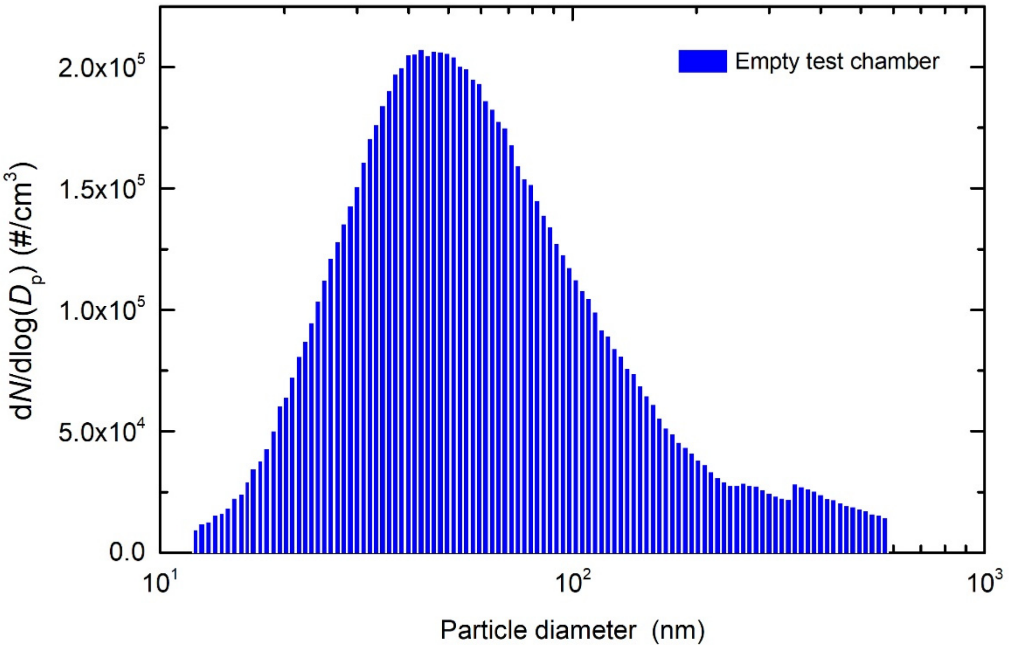


Figure 3S: The particle distribution (d$N$/dlog$D_P$) as a function of particle diameter $D_P$ inside an empty test chamber after stationary conditions have been achieved. Multiple charge correction and diffusion correction were used.

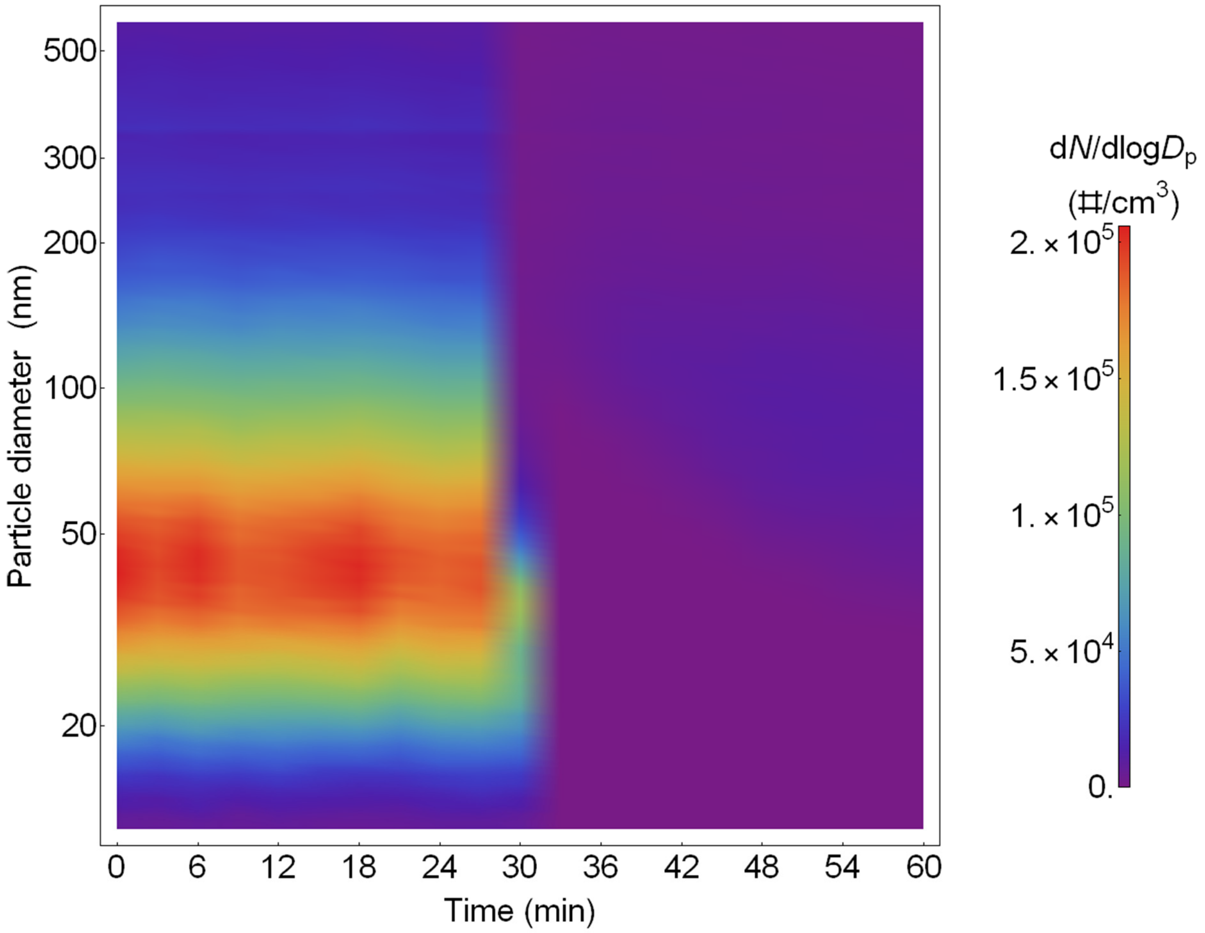


Figure 4S: (a) Normalized concentration (d*N*/dlog$D_p$ [#/cm³]) of NPs given as diameter in log scale vs time.

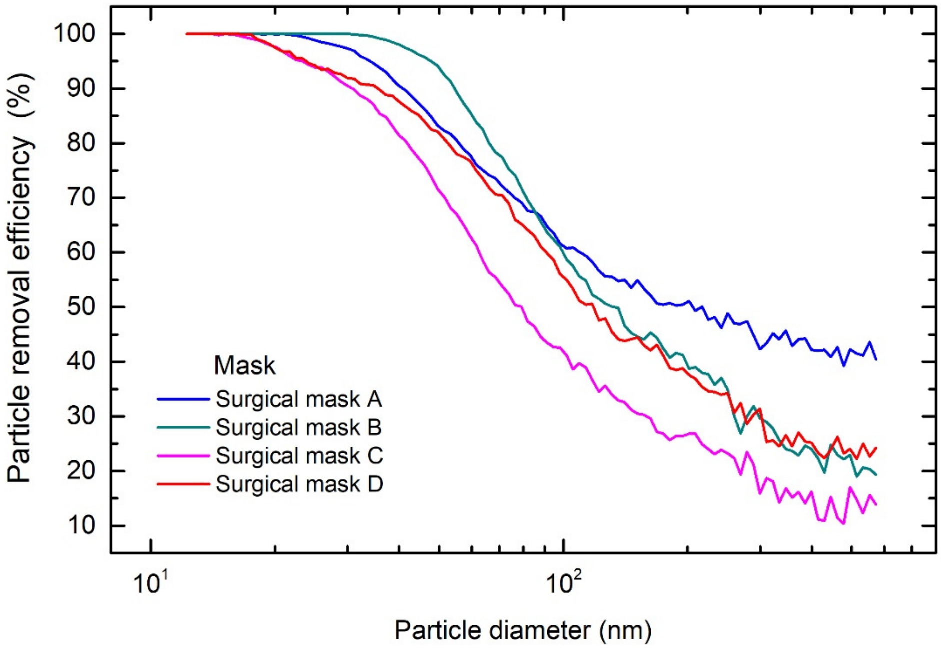


Figure 5S: Surgical masks, PRE as a function of particle diameter for different surgical masks.

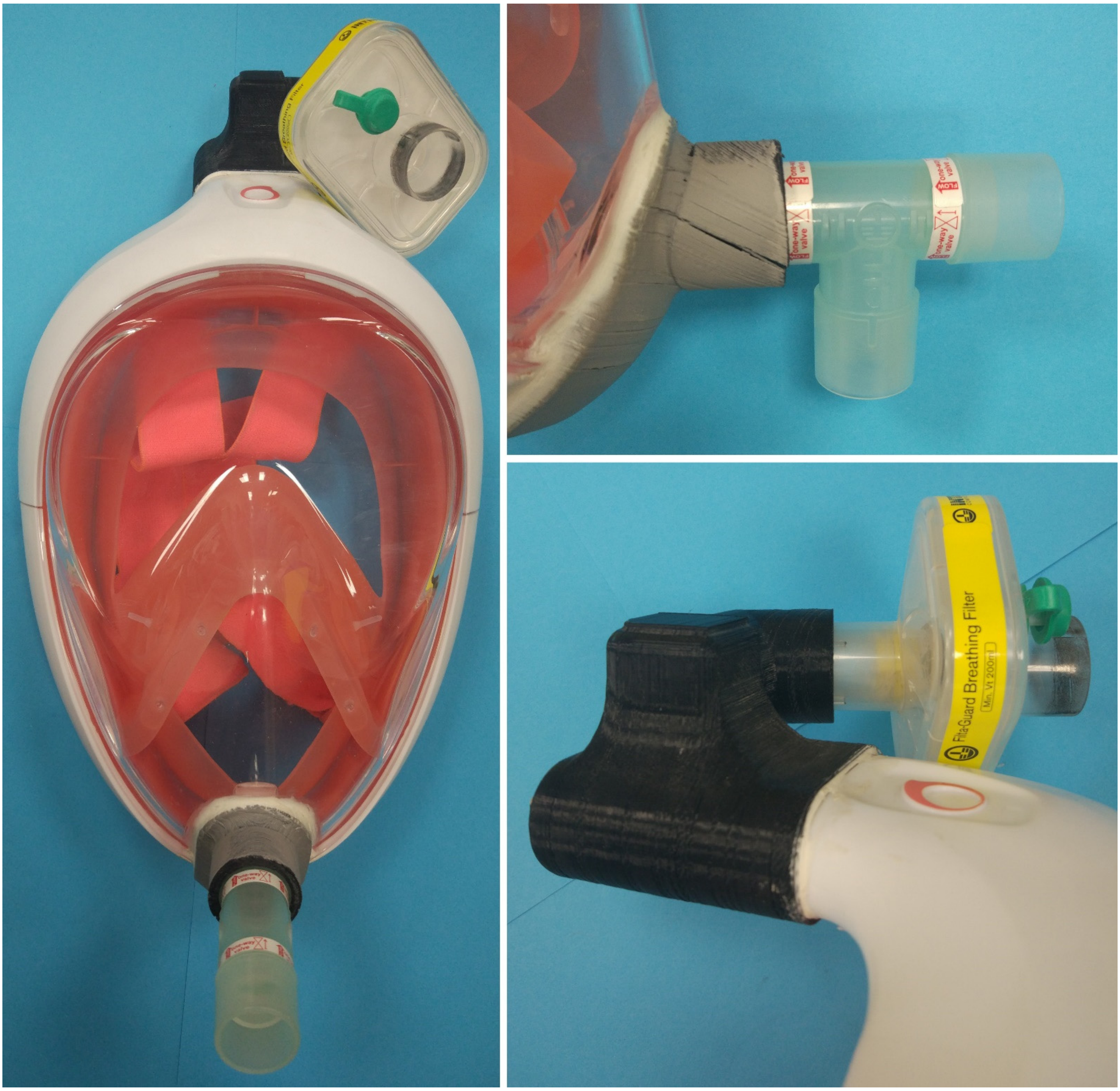


Figure 6S: Modified snorkelling mask.

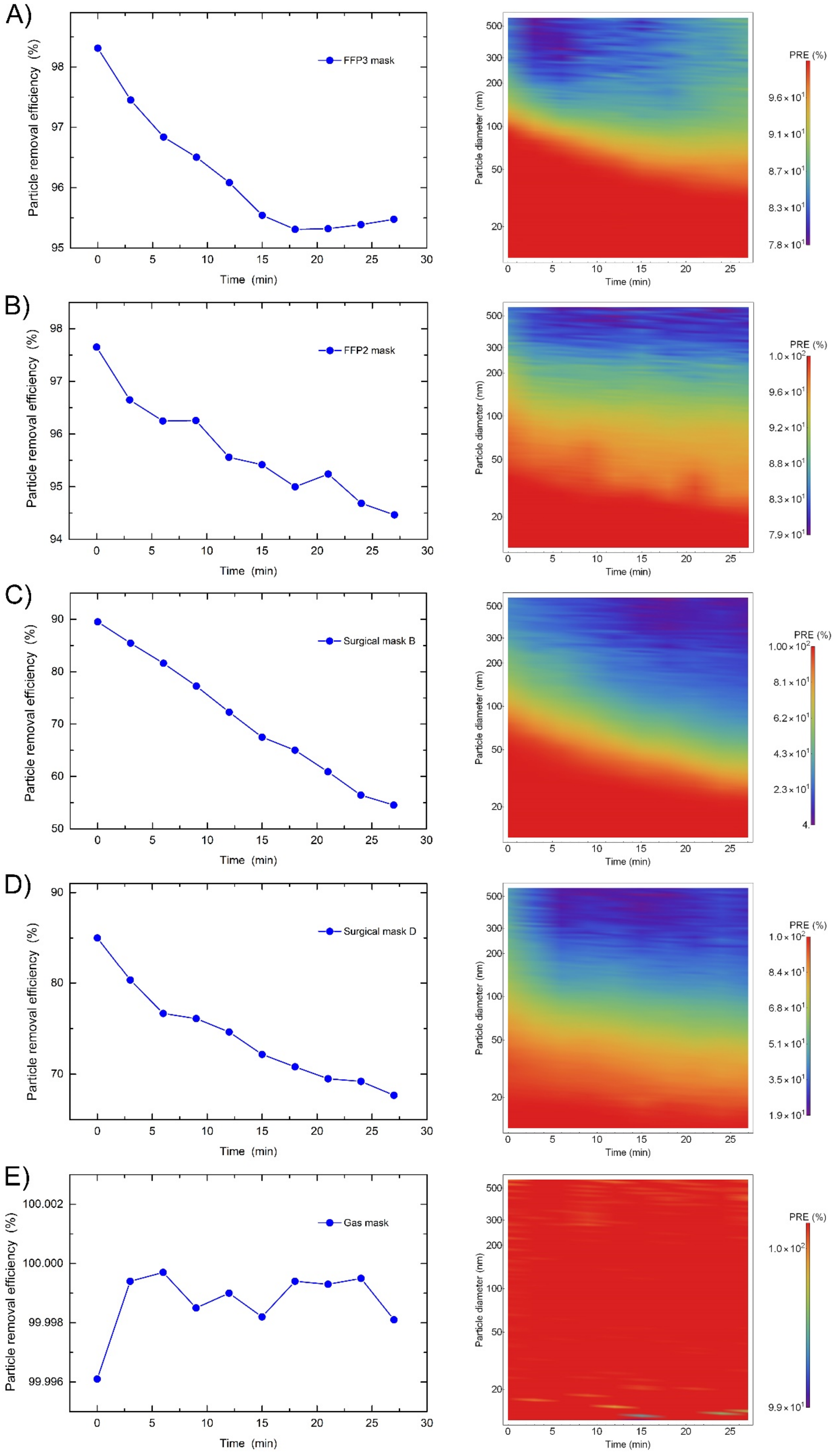


Figure 7S: Particle removal efficiency (PRE) as a function of time and PRE given as a particle diameter in log scale vs time of measurement plots for different masks.

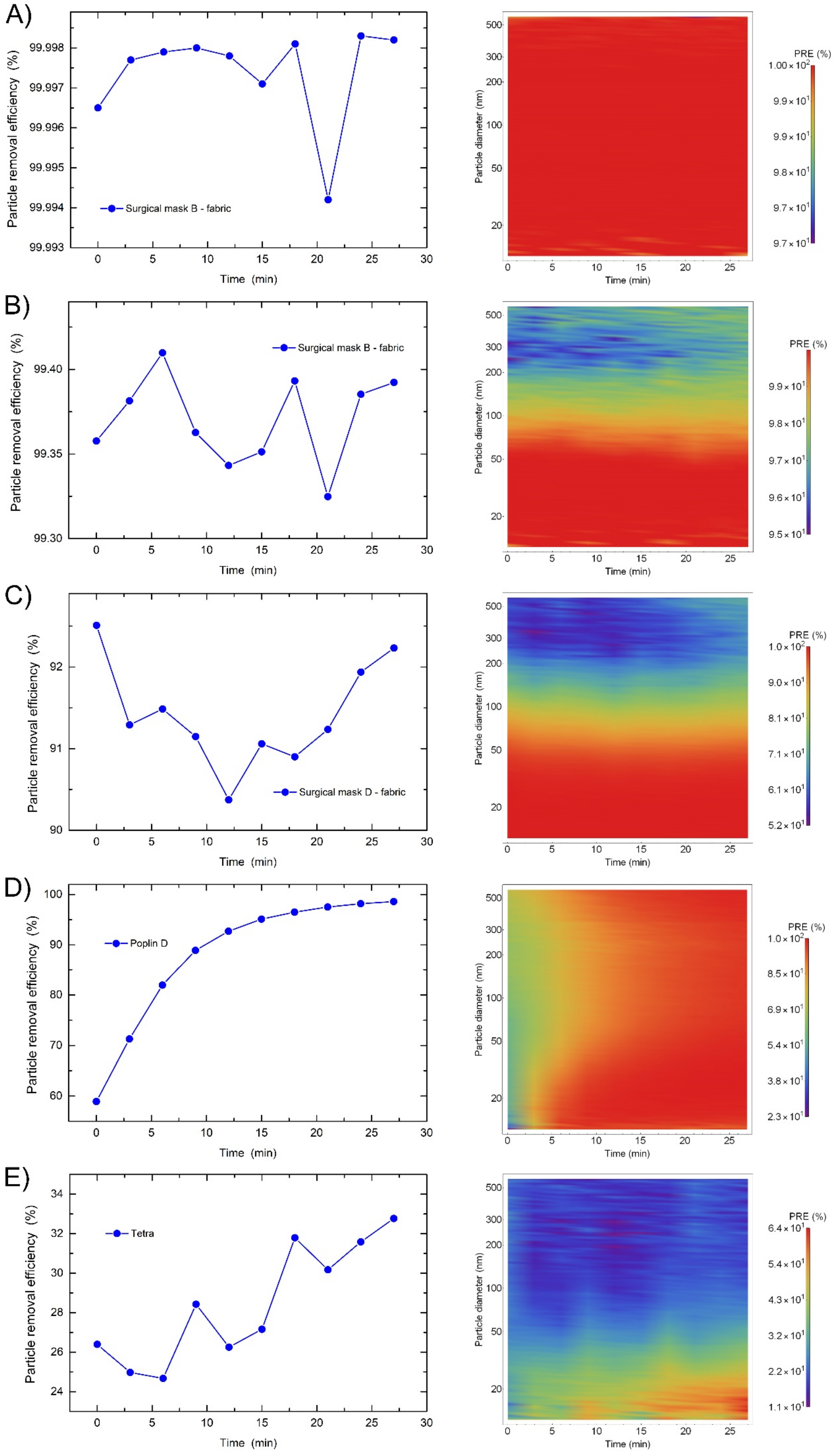


Figure 8S: Particle removal efficiency (PRE) as a function of time and PRE given as a particle diameter in log scale vs time of measurement plots for different fabrics.

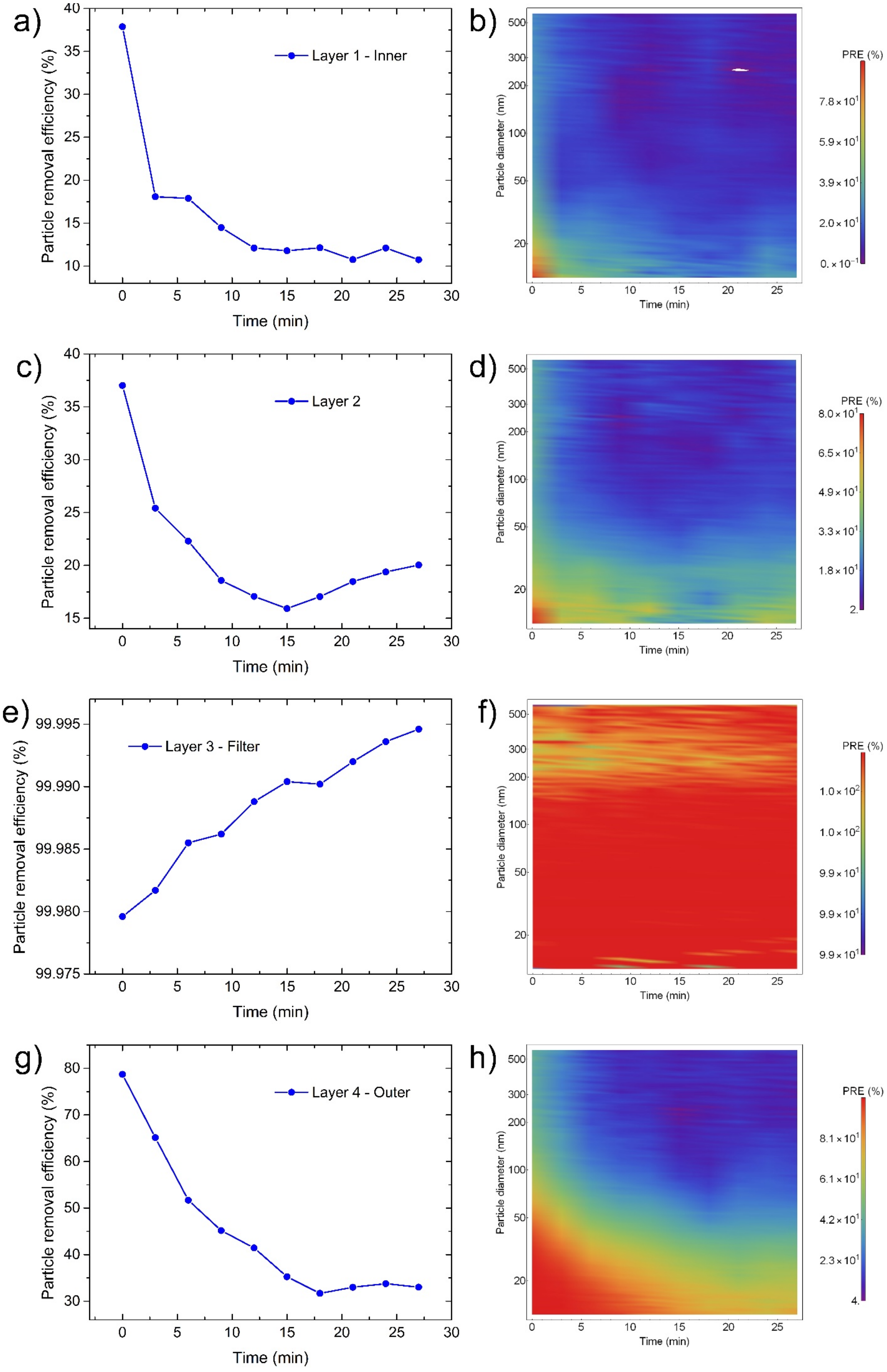


Figure 9S: Particle removal efficiency (PRE) as a function of time and PRE given as a particle diameter in log scale vs time of measurement plots for different layers of the FFP2 mask.

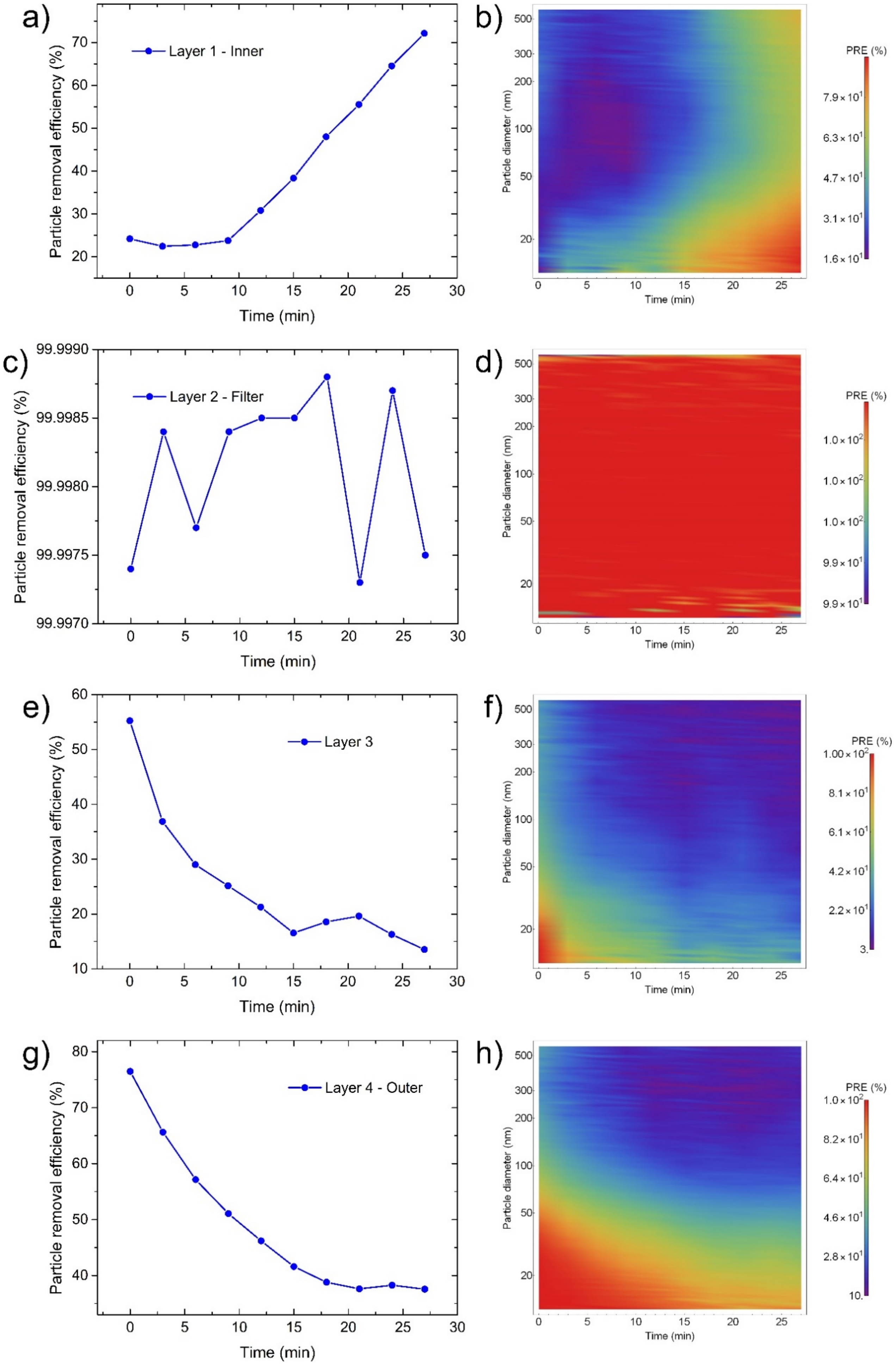


Figure 10S: Particle removal efficiency (PRE) as a function of time and PRE given as a particle diameter in log scale vs time of measurement plots for different layers of the FFP3 mask.
